# Relationships between nitrogen-fixing bacteria community structure in *Vicia villosa* nodules, soil properties and rocky desertification degree in karst area southwest China

**DOI:** 10.1371/journal.pone.0329408

**Published:** 2025-08-01

**Authors:** Yuanyuan Qi, Yating He, Li Yao, Qiuli Yan, Chengyi Wu, Yunpeng Wu, Jinhua Wang

**Affiliations:** 1 College of Biological Science and Food Engineering, Southwest Forestry University, Kunming, China; 2 College of Dai Medical, West Yunnan University of Applied Sciences, Jinghong, Yunnan, China; Nepal Agricultural Research Council, NEPAL

## Abstract

Rocky desertification, a common phenomenon in karst ecosystems, significantly impacts soil fertility and vegetation restoration. Therefore, understanding the relationship between nitrogen-fixing bacteria and soil properties under different degrees of rocky desertification is crucial. Our experiment was conducted to investigate the bacterial community structure and the main environmental factors affecting the distribution of the nitrogen-fixing bacteria in the nodules of *V. villosa*. Based on *nifH* gene sequence analysis, we found that the community composition of nitrogen-fixing bacteria in the nodules was significantly correlated with the degree of rocky desertification. The soil physicochemical properties affecting community composition were analyzed. The results revealed that: (1) The soil water content, alkali-hydrolyzable nitrogen content, and total nitrogen content in the slight rocky desertification area GJ4 were significantly higher than those in the moderate rocky desertification areas KY and MZ3. (2) There were significant differences in the community composition of nitrogen-fixing bacteria across the four rocky desertification areas (R^2^ = 0.448, P = 0.001). Within the same area, the Shannon index in slight rocky desertification was significantly higher than that of moderate rocky desertification. Rhizobium was the dominant genus. (3) In Gejiu, Yunnan Province, it has been observed that there is a clear negative correlation was observed among rocky desertification grade, soil water content, and nitrogen-fixing bacterial diversity in the typical karst ecosystem. Specifically, intensifying rocky desertification significantly reduces soil moisture and bacterial diversity. The degree of soil rocky desertification, total nitrogen content, total phosphorus content, and pH of soil are the main factors that play a key role in the community composition of the nitrogen-fixing bacteria in the nodules of *V. villosa*. This study provides a theoretical basis for the control of rocky desertification.

## Introduction

Rocky desertification, a severe form of land degradation, involves soil erosion, rock exposure, destruction of land productivity, and overall deterioration in fragile karst regions [1,2]. Karst landscapes, found across the globe, cover an estimated 50 million km^2^ in total area [3]. China, known for having one of the widest distributions and largest areas of karst worldwide, features 1.37 million km^2^ of exposed karst landscapes, which constitute approximately 13% of the country's total land surface [4]. Yunnan Province has the second largest area of rocky desertification in southwest China after Guizhou Province. It is characterized by a highland region where carbonate rocks are concentrated and contiguous, along with a distinctive climate and a less developed economy. All these factors contribute to its more severe rocky desertification issues. The interaction between karst landform conditions and human socioeconomic activities leads to vegetation destruction, severe soil erosion, and an increasing rate of rock exposure. These side effects result in a karst rocky desertification ecosystem that exhibits significant vulnerability and the phenomenon of rocky desertification.

Karst rocky desertification is the most severe ecological and geological environmental problem in southwest China, significantly hindering both environmental quality and socioeconomic development in the region. This issue has also led to a drastic reduction in biomass, affecting the ecological balance of the area [1,5,6]. Rocky desertification has caused significant alterations in the species composition and physiology within plant communities, impacting biodiversity and ecosystem health. Rocky desertification is directly associated with a range of ecological degradation outcomes, including exacerbated soil erosion, severe water and soil loss, extensive bedrock exposure, and significant land degradation [7]. To address the issue of rocky desertification, ecological restoration initiatives, including the protection of natural forests and the conversion of farmland back to forest, have been put into practice in the karst regions of southwest China [8,9]. The expansion of rocky desertification adversely affects ecosystem productivity and the sustainability of socioeconomic development, while also garnering increasing attention from both the public and the scientific community [10]. Accordingly, studying the control of karst rocky desertification is crucial for achieving ecological restoration and sustainable agricultural development.

Previous studies have shown that changes in rocky desertification in the southwest region significantly affect soil properties, which in turn influence the structure and function of microbial communities. [11–13]. Soil, a fundamental and crucial factor in shaping phytocommunity structure and providing nutrients to plants in terrestrial ecosystems, greatly influences vegetation cover, particularly in areas affected by rocky desertification [14–16]. Soil properties show significant differences in total nitrogen, pH, total potassium content, and the nitrogen-fixing bacterial community among different grades of rocky desertification [17]. Potential relationships may exist between communities of nitrogen-fixing bacteria, soil properties, and vegetation coverage within karst ecosystems [5]. Microorganisms are involved in the biochemical reactions that occur during both vegetation and soil restoration processes. Controlling karst rocky desertification largely depends on vegetation restoration, and microorganisms, especially nitrogen-fixing bacteria, play a vital role in either directly or indirectly enhancing plant growth [18–20].

Nitrogen-fixing bacteria are a group of bacteria that reside in the soil and can convert atmospheric nitrogen into ammonia that plants can directly utilize [21,22]. Legumes form nodules with *Rhizobia* and effectively fix nitrogen in the air [22,23]; *Rhizobium* and *Baldaniorum* strains were co-inoculated in Legumes to enhance their salt tolerance [24]. Research on endophytic bacteria, including nitrogen-fixing bacteria, in extreme environments aims to foster plant growth and stress resistance.

*V. villosa* is a species of annual or perennial herbaceous plants in the family Leguminosae [25]. It is characterized by its wide adaptability, strong resistance, well-developed root system, robust main root, and high nitrogen content [26,27], and is also a green manure crop. High-throughput sequencing was employed to evaluate the nitrogen-fixing bacteria of *V. villosa*. The studies revealed that the nitrogen-fixing bacteria extracted from crops’ nodules in arid regions demonstrated strong temperature adaptability, drought tolerance, and adaptability to various environmental factors [28,29]. Current research on the nitrogen-fixing bacteria in nodules of *V. villosa* in rocky desertification areas is limited.

The karst area in Yunnan Province constitutes 28.1% of the province's total land area, ranking it among the most extensively karstified provinces in China [30,31]. The total karst area encompasses 8 counties, and cities including Mengzi, Gejiu, Kaiyuan, and Jianshui in Honghe Prefecture constitute over 53% of the province's total land area [32]. Currently, research on *V. villosa* both domestically and internationally is primarily concentrated in the areas of feed and fertilizer. Simultaneously, the ecological restoration potential of *V. villosa* is only beginning to be realized, offering significant opportunities for further development and utilization. However, there is limited study available regarding the community composition of the nitrogen-fixing bacteria in *V. villosa* nodules in Yunnan Province’s rocky desertification areas and their correlation with soil physiochemical properties [33]. The *nifH* gene, which encodes an iron-containing nitrogenase protein, is widely found in nitrogen-fixing microorganisms. Therefore, the *nifH* gene is an ideal genetic marker for studying these microorganisms. In this study, *V. villosa* from the rocky desertification area of Yunnan Province served as the experimental plant. High-throughput sequencing of the *nifH gene* was conducted on the nitrogen-fixing bacteria of *V. villosa* nodules, along with analyses of α diversity, β diversity, species composition, species differences, and environmental factor associations. Concurrently, eight physical and chemical indexes including total nitrogen (TN), alkali-hydrolyzable nitrogen (AN), total phosphorus (TP), available phosphorus (AP), total potassium (TK), available potassium (AK), soil water content (SWC) and pH of the rhizosphere soil were assessed as environmental factors. A comprehensive analysis has examined the relationship between the soil physicochemical properties and the community composition of the nitrogen-fixing bacteria in *V. villosa* nodules from Yunnan Province’s rocky desertification areas. This analysis provides a theoretical basis for developing strategies to improve soil quality during rocky desertification control efforts. Soil physicochemical properties, by redistributing nutrients among microorganisms, influence the structure of microbial communities and enhance microbial abundance; nitrogen-fixing bacteria are particularly key in soil improvement [34–36]. However, our comprehensive understanding of the interactions between the nitrogen-fixing bacteria in aboveground plants and soil properties remains limited [37,38]. Therefore, studying how soil physicochemical properties affect the diversity and function of the nitrogen-fixing bacteria communities in the host plants’ nodules is of great significance. This study aims to explore the relation between soil physicochemical properties and the diversity and function of nitrogen-fixing bacteria communities in plant nodules. Are there significant differences in soil physicochemical properties, nitrogen-fixing bacterial diversity, and community composition across different rocky desertification areas? Does soil physicochemical properties have a significant impact on nitrogen-fixing bacteria?

## Materials and methods

Root nodules of *V. villosa* were collected from four rocky desertification areas in China, Gejiu1 (GJ1), Gejiu4 (GJ4), Mengzi3 (MZ3), Kaiyuan2 (KY2), respectively.

According to the research by Dai Quanhou and other scholars, when the bedrock exposure rate is 30%−50%, it is classified as mild rocky desertification; when it is 50%−70%, it is moderate rocky desertification; and when it is 70%−100%, it is severe rocky desertification [39,40]. This study uses this classification standard to divide the grades of rocky desertification. Among these areas, GJ1, MZ3, and KY2 represent moderate rocky desertification (MRD), while GJ4 represents slight rocky desertification (SRD). Biological replication was performed three times for each group of samples, resulting in a total of twelve samples. The sampling information is detailed in the S1 Table.

The whole plant of *V. villosa* was dug up with a sterile iron shovel, and the samples of *V. villosa* with a higher number of nodules, full shape, and large volume were selected. The roots of different samples were placed into sterile storage tubes and stored in a liquid nitrogen container, transported back to the laboratory. Rinse the soil from the root nodules with tap water, then rinse them with sterile water, and soak them completely in sterile water until they are fully imbibed. The root nodules were first blotted dry with filter paper and then soaked in 95% ethanol for 5 minutes. Afterward, they were removed and placed into a 0.1% mercuric chloride solution for surface sterilization for 3 minutes. The nodules were then washed five times with sterile water, and the final wash was spread onto YMA and NA media before being incubated at 28°C for 4 days. If no colonies form, it indicates that the root nodules have been successfully surface-disinfected. The surface-disinfected nodules were then placed into centrifuge tubes containing 0.5 mL of 0.85% NaCl solution for the total DNA extraction of the bacteria within 2 days [41–43]. To obtain rhizosphere soil, we first removed the large hardened soil from the roots of *V. villosa*. Next, we collected the soil that was attached to the root surface within the 0–2 mm layer. The soil was then enclosed in sterile bags for the determination of the soil's physicochemical properties [44].

### Determination of soil environmental factors

The soil physicochemical properties were assessed after natural drying and passing through a 2 mm sieve to remove pebbles, roots, and other debris. The soil pH was determined using a pH meter (PHS-3C) (water: soil = 2.5: 1). The Kjeldahl method was employed to measure the TN. The content of AN was calculated with the diffusion absorption method. TP was quantified using molybdenum antimony anti-colorimetric UV spectrophotometry, and the extraction method was used to determine the amount of AP. SWC was established by the constant weight method. TK was determined using the NaOH melting-flame photometric technique, and AK was measured using the ammonium acetate extraction-flame photometric method. For each sample, three biological replicates were set up. The data were analyzed using SPSS 25.0 software. The mean and standard error were used to express the statistical data. Significant differences were noted by *P* < 0.05 and highly significant differences were noted by *P* < 0.01.

### High-throughput sequencing of nitrogenase *nifH* gene

The *nifH* gene primers [45](*nifH* F: AAAGGYGGWATCGGGYAARTCCACCAC; *nifH* R: TTGTTSGCSGCRTACATSGCCATCAT) were used to amplify the *nifH* gene region. PCR amplification conditions: 95°C for 3 minutes, followed by 30 cycles of 95°C for 30 seconds, 60°C for 30 seconds, 72°C for 42 seconds, and finally 72°C for 10 minutes. The PCR reaction was performed in triplicate and analyzed using a 2% agarose gel electrophoresis.

The PCR products were identified and quantified using the QuantiFluor™ ST Blue fluorescence quantitation system (Promega Corporation), and Illumina library preparation and sequencing were conducted based on the preliminary quantification from electrophoresis.

### Bioinformatics analysis

Normal distribution and equal variance tests were used for all data. Single-factor analysis of variance (ANOVA) was used to evaluate whether the change in rocky desertification had significant effects on the nitrogen-fixing bacteria community and soil physicochemical properties. Details are shown in S2 Table and S3 Table. If the effects were found to be significant, the least significant difference (LSD) test was used to compare the mean values of all soil parameters (*P* < 0.05). After extracting genomic DNA from the samples, the V5-V7 region of the 16S rDNA was amplified using specific primers with barcodes. The primer sequences were: 799F: AACMGGATTAGATACCCKG; 1193R: ACGTCATCCCCACCTTCC. After the purified amplification product was ligated to the sequencing adapter, the sequencing library was constructed and subsequently sequenced on an Illumina platform. After obtaining raw reads from sequencing, the Usearch software was employed. Low-quality reads were eliminated initially, then double-ended reads were assembled into tags, and subsequently, the low-quality tags were removed. The resulting data were referred to as Clean Tags. Next, based on the Clean Tags, OTU clustering was conducted on the QIIME platform for non-repeating sequences, excluding single occurrences, at a 97% similarity threshold. During the clustering process, chimeric sequences were identified and removed, and a representative OTU sequence was selected and tabulated. To obtain the species classification information of each OTU, the Ribosomal Database Project (RDP) Classifier Bayesian algorithm was used for analyzing the representative sequences of each OTU with 97% similarity, and species annotations and abundance information were generated at each classification level. Using OTU sequence and abundance data, we conducted species annotation, species composition analysis, indicator species analysis, α diversity analysis [46,47], β diversity analysis, and community function prediction. α diversity analysis is calculated as follows:

Chao Index: The Chao index, estimated using the Chao1 algorithm, is a measure used to estimate the number of OTUs (Operational Taxonomic Units) in a sample. Commonly employed in ecology to estimate the total number of species, the Chao1 index was first proposed by Chao.

The formula used in this analysis is as follows:


$$S_{chao1}=S_{obs}+\frac{n_{1}(n_{1}-1)}{2(n_{2}+1)}$$

$S_{chao1}$ = The estimated number of OTUs;

$S_{obs}$ = The actual number of observed OTUs;

$n_{1}$ = The number of OTUs containing only one sequence (i.e., “singletons”);

$n_{2}$ = The number of OTUs containing only two sequences (i.e., “doubletons”)。

ACE Index: The ACE (Abundance-based Coverage Estimator) index is used to estimate the number of OTUs (Operational Taxonomic Units) in a community. Proposed by Chao, it is one of the commonly used indices for estimating the total number of species in ecology and differs from the Chao1 algorithm.

The formula used in this analysis is as follows:


$$S_{ACE}=\left\{ \begin{array}{c} S_{abund}+\frac{S_{rare}}{C_{ACE}}+\frac{n_{1}}{C_{ACE}}{\overset{\wedge 2}{\gamma }}_{ACE},for\;{\overset{\wedge }{\gamma }}_{ACE}<0.08\\ S_{abund}+\frac{S_{rare}}{C_{ACE}}+\frac{n_{1}}{C_{ACE}}{\overset{\sim 2}{\gamma }}_{ACE},for\;{\overset{\wedge }{\gamma }}_{ACE}\geq 0.08 \end{array}\right.$$

where:


$$N_{rare}=\sum_{i-1}^{abund}in_{i},C_{ACE}=1-\frac{n_{1}}{N_{rare}}$$

$${\overset{\wedge 2}{\gamma }}_{ACE}=\max\left[\frac{S_{rare}}{C_{ACE}}\frac{\sum {\mathrm{\backslash nolimits}}_{i-1}^{abund}i(i-1)n_{i}}{N_{rare}(N_{rare}-1)}-1,0\right]$$

$${\overset{\sim 2}{\gamma }}_{ACE}=\max\left[{\overset{\wedge 2}{\gamma }}_{ACE}\left\{1+\frac{N_{rare}(1-C_{ACE})\sum_{i-1}^{abund}i(i-1)n_{i}}{N_{rare}(N_{rare}-C_{ACE})}\right\},0\right]$$

$n_{i}$ = Number of OTUs with 1 sequence;

$S_{rare}$ = Number of OTUs with “abund” sequences or fewer;

$S_{abund}$ = Number of OTUs with more than “abund” sequences;

abund = The threshold for “abundant” OTUs, default is 10.

Simpson Index: One of the indices used to estimate microbial diversity in a sample, proposed by Edward Hugh Simpson in 1949. It is commonly used in ecology to quantitatively describe biodiversity in a given area. A higher Simpson index value indicates lower community diversity.


$$D_{simpson}=\frac{\sum {\mathrm{\backslash nolimits}}_{i=1}^{S_{obs}}n_{i}(n_{i}-1)}{N(N-1)}$$

where:

$S_{obs}$ = Actual number of observed OTUs;

$n_{i}$ = Number of sequences in the i-the OTU;

N = Total number of sequences.

Shannon Index: One of the indices used to estimate microbial diversity in a sample. Similar to the Simpson diversity index, it is often used to reflect community alpha diversity. A higher Shannon value indicates higher community diversity.


$$H_{shannon}=-\sum_{i=1}^{S_{obs}}\frac{n_{i}}{N}\ln\frac{n_{i}}{N}$$

where:

$S_{obs}$ = Actual number of observed OTUs;

$n_{i}$ = Number of sequences in the i-the OTU;

N = Total number of sequences.

Coverage: Refers to the coverage of the libraries in each sample. The higher the value, the higher the probability that sequences in the sample have been detected, and the lower the probability that they have not been detected. This index reflects whether the sequencing results represent the true situation of microorganisms in the sample.


$$C=1-\frac{n_{2}}{N}$$

where:

$n_{2}$ = Number of OTUs containing only one sequence;

N = Total number of sequences in the sample.

If valid groups are identified, comparisons and statistical tests are performed to assess the differences among them. Finally, we utilized R software (version 3.3.1) to perform a Spearman analysis, generate a heatmap, and used Canoco 5.0 software to examine the relationships between soil physicochemical properties and the microbial communities.Based on *nifH* gene sequencing data, we conducted an analysis using the PICRUSt2 functional prediction software. In conjunction with the Integrated Microbial Genomes database, we compiled high-quality bacterial and archaeal genomes and constructed a phylogenetic tree based on these sequences for functional prediction. All the data were processed using Excel 2010 to elucidate the relationship between microorganisms and the environment.

The raw sequence data presented in this article have been deposited in the NCBI platform [PRJNA945808], which can be reached by the below links https://www.ncbi.nlm.nih.gov/sra/PRJNA945808

## Results

### Determination of environmental factors

The pH, TN, TP, and other five environmental factors of the rhizosphere soil collected from Gejiu, Kaiyuan, and Mengzi were determined. As shown in Table 1: (1) The SWC in SRD was significantly higher than that in MRD, being 1.13 times, 1.11 times, and 1.17 times respectively. (2) The contents of TP were 1.40 times, 1.83 times, and 3.79 times higher in Mengzi than those in Gejiu and Kaiyuan districts. Additionally, the contents of AP in Mengzi were 4.87 times, 6.67 times, and 26.04 times greater than those in Gejiu and Kaiyuan districts. (3) The content of AN in GJ4 with SRD was 4.73 times and 2.99 times that of MRD areas in Mengzi and Kaiyuan, respectively. (4) Soil TN content in SRD was significantly higher than in MRD, being 3.2 times greater in Kaiyuan and 2.8 times greater in Mengzi. (5) In different rocky desertification areas, there were no significant differences in TK or soil pH. SRD areas exhibited significantly higher SWC, AN, and TN contents compared to MRD areas. The results indicated an inverse correlation between the SWC, AN, and TN contents in the rhizosphere soil of *V. villosa* and the severity of rocky desertification.

**Table 1:** Soil properties related to *V. villosa* in different rocky desertification grades.

Environmental factor	GJ1	GJ4	KY2	MZ3
pH	7.5 ± 0.03^a^	7.62 ± 0.04^a^	7.96 ± 0.14^a^	7.54 ± 0.25^a^
TN(g/kg)	0.76 ± 0.17^a^	0.50 ± 0.05^B^	0.24 ± 0.03^c^	0.27 ± 0.03^c^
TP(g/kg)	0.38 ± 0.04^b^	0.29 ± 0.04^b^	0.14 ± 0.07^c^	0.53 ± 0.11^a^
AN(mg/kg)	213.97 ± 29.13^a^	211.87 ± 19.86^a^	44.80 ± 28.31^B^	70.93 ± 33.78^B^
AK(mg/kg)	5.86 ± 0.93^a^	5.62 ± 0.10^a^	4.29 ± 0.27^B^	1.46 ± 0.08^c^
TK(mg/kg)	0.142 ± 0.00^a^	0.26 ± 0.00^a^	0.98 ± 1.41^a^	0.26 ± 0.00^a^
AP(mg/kg)	11.81 ± 1.85^B^	8.63 ± 0.42^B^	2.21 ± 1.49^c^	57.55 ± 6.28^a^
SWC (%)	13.89 ± 0.76^b^	15.82 ± 0.35^a^	14.25 ± 0.79^b^	13.49 ± 0.87^b^

Note: TN: Total nitrogen content in the soil. TP: Total phosphorus content in the soil. AN: Alkali-hydrolyzable nitrogen content. AP: Available phosphorus content. AK: Available potassium content. TK: Total potassium content in the soil. SWC: soil water content. This figure illustrates the variations in soil physicochemical properties across different regions. In the table, lowercase letters indicate a significant difference (*P* < 0.05), while uppercase letters indicate an extremely significant difference (*P* < 0.01).

### Diversity of the nitrogen-fixing bacteria in nodules of *V. villosa*

The α diversity index of the nitrogen-fixing bacteria in nodules of *V. villosa* is presented in **Table 2**. The Shannon index of the nitrogen-fixing bacteria in SRD was significantly higher than that of GJ1 in MRD, but no significant differences were detected when compared to Mengzi and Kaiyuan. The Simpson index of the nitrogen-fixing bacteria in the Kaiyuan area was significantly lower than those in the Gejiu and Mengzi areas. No significant differences were found in the Sobs index of the nitrogen-fixing bacteria of *V. villosa* between the four groups. The results suggest that the diversity of the nitrogen-fixing bacteria in the same area was inversely correlated with the degree of rocky desertification.

**Table 2:** Diversity metrics of the nitrogen-fixing bacteria in the root nodules of *V. villosa.*

Group	Sobs	Shannon	Simpson	Chao	ACE	Coverage
GJ1	27.67 ± 4.93^a^	0.89 ± 0.31^b^	0.61 ± 0.15^a^	30.22 ± 4.55a	31.09 ± 4.24^a^	0.9997
GJ4	23.67 ± 4.16^b^	1.04 ± 0.09^a^	0.42 ± 0.02^a^	28.10 ± 5.90^b^	37.18 ± 13.83^a^	0.9997
KY2	24.00 ± 3.61^b^	1.48 ± 0.35^a^	0.32 ± 0.10^b^	26.00 ± 3.50^b^	27.19 ± 5.81^a^	0.9998
MZ3	36.67 ± 10.12^a^	0.99 ± 0.34^a^	0.54 ± 0.17^a^	39.84 ± 8.67^a^	39.93 ± 7.99^a^	0.9997

Note: Sobs (Species Observed): The number of observed species within each sample. Shannon Index: A measure of diversity that takes into account both species richness and evenness. Simpson Index: A measure of diversity that emphasizes the dominance of the most abundant species. Chao Index: An estimator of species richness that accounts for the presence of rare species. ACE (Abundance-based Coverage Estimator): An estimator of species richness that combines abundance and incidence data. Coverage: A measure of how well the sample represents the true community. In the table, lowercase letters indicate a significant difference (*P* < 0.05).

### Relationship between environmental factors and the α diversity index of nitrogen-fixing bacteria

The correlation between the α diversity index of the nitrogen-fixing bacteria and environmental factors revealed that TP was strongly positively correlated with the Chao and Simpson indices, and strongly negatively correlated with the Shannon index (**Table 3**). AK exhibited a negative correlation with the Shannon index, whereas the Simpson index showed a positive correlation. Both the Sobs and Chao indices exhibited a very strong positive correlation with AP. The diversity of the nitrogen-fixing bacteria in *V. villosa* is anticipated to increase as soil AP content increases.

**Table 3:** Correlation between environmental factors and the α diversity index of nitrogen-fixing bacteria.

	pH	TN (g/kg)	TP (g/kg)	TK (g/kg)	AN (g/kg)	AP (g/kg)	AK (g/kg)	SWC (%)	Rocky desertification Degree (RD)
Sobs	−0.254	−0.093	0.536	0.516	−0.354	0.750**	0.045	−0.363	0.342
Shannon	0.427	−0.441	−0.600*	0.226	−0.532	−0.257	−0.690*	0.067	0.11
Simpson	−0.351	0.452	0.681*	−0.231	0.444	0.336	0.609*	−0.302	0.194
Chao	−0.249	−0.077	0.600*	0.451	−0.28	0.773**	0.15	−0.358	0.236
ACE	−0.22	0.006	0.421	0.313	−0.054	0.466	0.292	0.05	−0.22

Note: pH: Soil pH value. TN: Total nitrogen content in the soil (g/kg). TP: Total phosphorus content in the soil (g/kg). TK: Total potassium content in the soil (g/kg). AN: Alkali-hydrolyzable nitrogen content (g/kg). AP: Available phosphorus content (g/kg). AK: Available potassium content (g/kg). SWC: Soil water content (%). Rocky Desertification Degree (RD): Degree of rocky desertification. This table presents the correlation coefficients between various environmental factors and the α diversity indices of nitrogen-fixing bacteria in the root nodules of *V. villosa*. The α diversity indices include Sobs, Shannon Index, Simpson Index, Chao Index, and ACE. In the table, * indicates a significant correlation (*P* < 0.05), and ** indicates an extremely significant correlation (*P* < 0.01). Each value represents the correlation coefficient between the environmental factor and the corresponding α diversity index.

### Composition of the nitrogen-fixing bacteria in nodules of *V. villosa*

**Fig 1:**
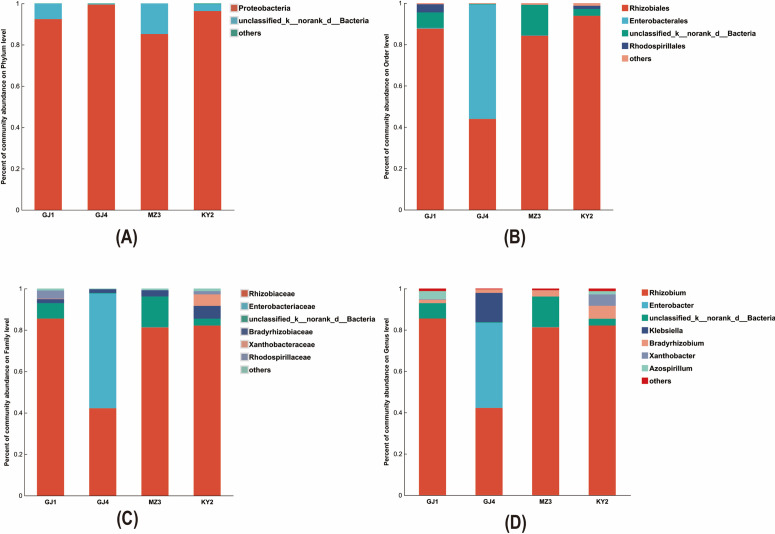
The species distribution stacking diagrams of the nitrogen-fixing bacteria within the nodules of *V. villosa.* (A) Phylum Level: Shows the distribution of nitrogen-fixing bacterial communities at the phylum level among different sites (GJ1, GJ4, MZ3, KY2). (B) Order Level: Illustrates the composition of nitrogen-fixing bacterial communities at the order level. (C) Family Level: Provides a breakdown of the bacterial communities at the family level. (D) Genus Level: Details the distribution at the genus level of nitrogen-fixing bacteria.

Illumina MiSeq was used to sequence the *nifH* gene. A total of 173157 valid reads were yielded from the 12 samples, and 165 operational taxonomic units (OTUs) were obtained by OTU division and clustering, which annotated to 6 phyla, 9 classes, 12 orders, 15 families, and 19 genera. At the phylum level (Fig 1A), the nitrogen-fixing bacteria were classified into Proteobacteria, Chlorophyta, an unclassified bacterial phylum, Firmicutes, and Cyanobacteria. Among these, Proteobacteria represented 92.43%, 99.48%, 85.21%, and 96.34% in GJ1, GJ4, MZ3, and KY2 respectively, and was the dominant phylum among the nitrogen-fixing bacteria in the nodules of *V. villosa*.

The nitrogen-fixing bacteria of *V. villosa* were categorized into 12 orders at the order level (Fig 1B), with Rhizobiales, Enterobacterales, and Rhodospirillales among them. In MRD samples, the dominant order Rhizobiales accounted for 87.67%, 84.28%, and 93.87% in GJ1, KY2, and MZ3 respectively. However, in SRD, this proportion was merely 43.97%. This indicates that the relative abundance of Rhizobiales among the nitrogen-fixing bacteria of *V. villosa* declined as the severity of rocky desertification decreased. Moreover, Enterobacterales were the predominant order, constituting 55.41% of the nitrogen-fixing bacteria in SRD.

At the family level (Fig 1C), the nitrogen-fixing bacteria in *V. villosa* were annotated to 15 families, including Rhizobiaceae, Enterobacteriaceae, Bradyrhizobiaceae, Xanthobacteraceae, and Rhodospirillaceae. The proportion of Rhizobia in GJ1, GJ4, KY2, and MZ3 were 85.51%, 42.23%, 81.28%, and 82.13%, respectively. The proportions of Bradyrhizobium were 1.92%, 1.75%, 3.00%, and 6.22%, respectively. The relative abundance of Enterobacteriaceae in GJ4 was 55.41%, rendering it the predominant family of nitrogen-fixing bacteria in *V. villosa* in SRD. The dominant family of the nitrogen-fixing bacteria in *V. villosa* in MRD was Rhizobiaceae.

At the level of genus (Fig 1D), 19 genera in total, including *Rhizobium*, *Pseudomonas*, *Klebsiella*, *Xanthobacter*, *Skermanella*, *Sinorhizobium*, *Pantoea*, *Paenibacillus*, *Enterobacter*, *Bradyrhizobium* and *Azospirillum*, were identified in the nitrogen-fixing bacteria within *V. villosa*. *Rhizobium* constituted 85.51%, 42.23%, 81.26%, and 82.13% of the bacterial communities in GJ1, GJ4, MZ3, and KY2, respectively. Additionally, *Enterobacter represented* 41.28% of the bacterial community in the SRD area. *Rhizobium* was identified as the predominant genus among the nitrogen-fixing bacteria in *V. villosa* nodules across rocky desertification areas.

Based on the analysis, *Rhizobium* emerges as the dominant genus across the four rocky desertification areas. This dominance may be attributed to *Rhizobium*’s nitrogen-fixation ability, enabling it to acquire additional nutrients and thus adapt to harsh environments. Furthermore, *Enterobacter* is more prevalent in SRD, possibly because it is better adapted to survive in areas with a lower degree of rocky desertification.

### Community composition difference of the nitrogen-fixing bacteria

**Fig 2:**
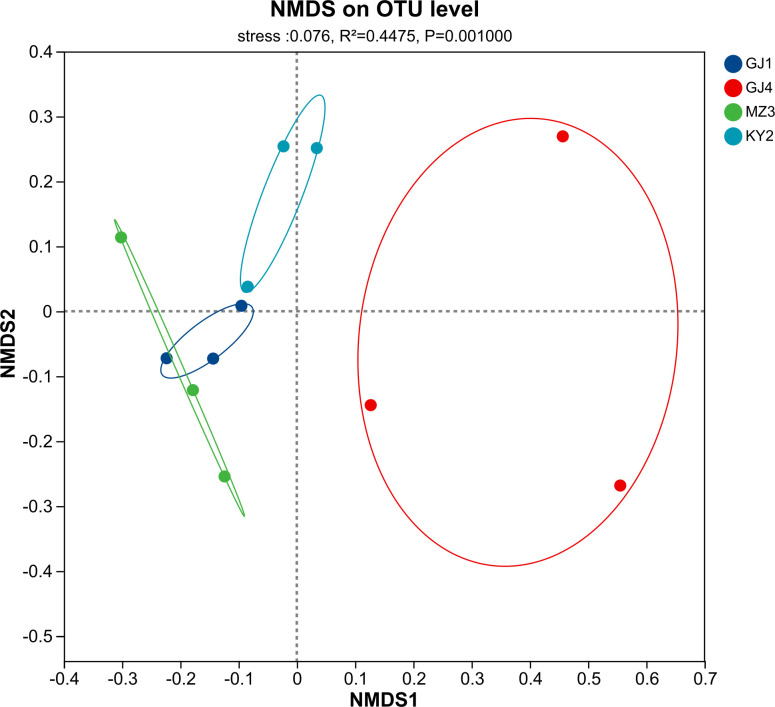
Non-metric Multidimensional Scaling (NMDS) analysis of nitrogen-fixing bacterial communities. Different colors represent samples from various sites: blue for GJ1, red for GJ4, green for MZ3, and cyan for KY2. The statistical results reveal a significant separation among the groups, with a stress value of 0.076, R^2^ = 0.4475, and P = 0.001000. The ellipses depict the 95% confidence ellipses for each group, indicating the spread and clustering of the samples within each group.

The β diversity principal coordinate analysis (NMDS) revealed whether the composition of the nitrogen-fixing bacteria from four distinct areas was similar or not. This NMDS (Fig 2) analysis chart illustrates the differences in bacterial community structure among samples at the OTU level. The stress value shown in the chart is 0.076, which is an indicator of the goodness-of-fit of the NMDS analysis. The lower the stress value, the better the model fits the data. It can be seen from the chart that the GJ4 samples have significant differences in bacterial community structure compared to the other sample groups (GJ1, MZ3, KY2). The GJ4 group samples exhibit a relatively consistent community structure. The other groups (GJ1, MZ3, KY2) also show a certain degree of consistency in their community structures, but the differences among them are relatively small. The P-value is 0.001, indicating the statistical significance of the differences between the samples. A P-value less than 0.05 is generally considered statistically significant, suggesting that there are significant differences in the community structures between the different sample groups. Overall, this analysis demonstrates the differences in bacterial community structure among various samples and points out that there are significant differences in community structure among the sample groups. The results indicated the degree of rocky desertification significantly influenced the community composition of the nitrogen-fixing bacteria across the four regions.

### Environmental factors’ impacts on the community structure of the nitrogen-fixing bacteria

**Fig 3:**
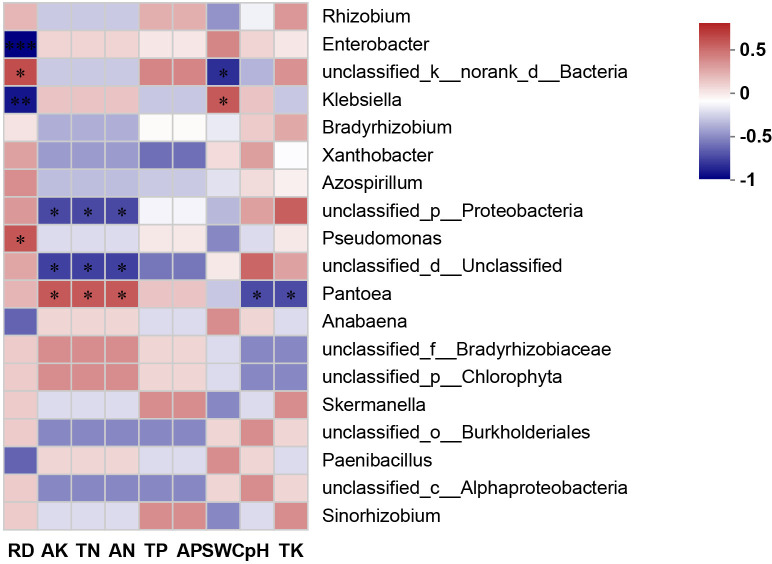
Heat map of soil physicochemical properties and the nitrogen-fixing bacteria in the root nodules of V. villosa. Color Gradient: Correlation coefficient ranging from −1 (strong negative) to 0.5 (strong positive).Significance:* indicates a significant correlation at the P < 0.05 level; ** indicates an extremely significant correlation at the P < 0.01 level.

The relationship between the top 20 nitrogen-fixing bacteria and soil environmental factors was analyzed using the Spearman correlation coefficient. As illustrated in the correlation heatmap (Fig 3), *Pantoea* demonstrated a significant positive correlation with AK, TN, and AN, while exhibiting a negative correlation with pH and TK. *Enterobacter* and *Klebsiella* presented a strong negative correlation with the RD, in contrast, *Pseudomonas* showed a significant positive correlation*. Klebsiella* also manifested a positive correlation with SWC. The majority of bacterial species were closely and significantly related to the degree of soil rocky desertification, suggesting that this factor exerted the most prominent influence on the community composition of the nitrogen-fixing bacteria among the various environmental parameters.

**Fig 4:**
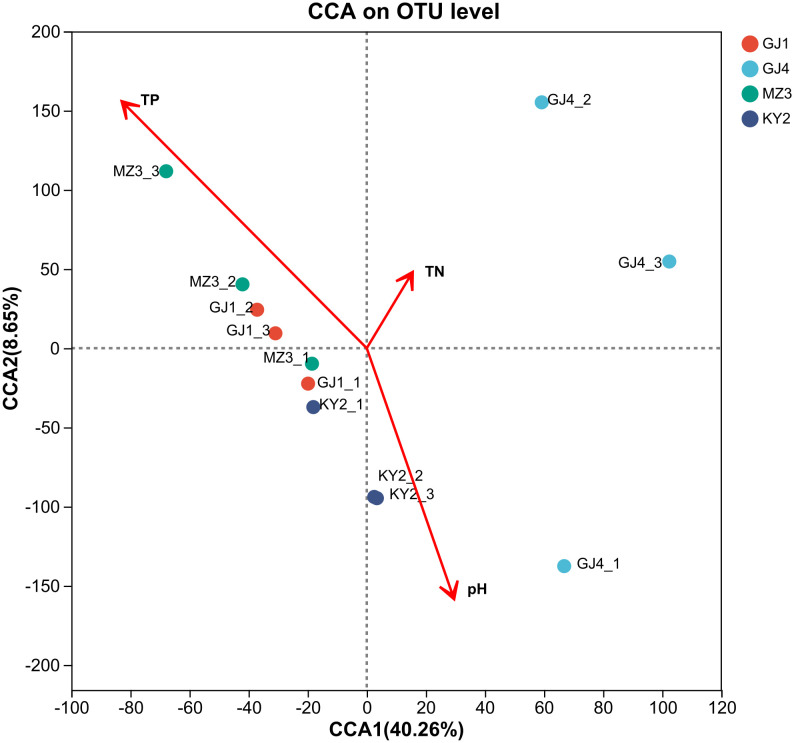
Canonical Correspondence Analysis (CCA) of soil physicochemical properties and the nitrogen-fixing bacteria in the root nodules of V. villosa. Sample Points: Different colors represent samples from various sites (GJ1, GJ4, MZ3, KY2). Environmental Factors: Arrows indicate the direction and strength of the relationship between soil properties and bacterial communities. CCA1 (40.26%): Explains 40.26% of the total variance in the community structure; CCA2 (8.65%): Explains 8.65% of the total variance. Ellipses: Represent the 95% confidence ellipses for each group, indicating the spread and clustering of the samples within each group.

The association between environmental factors and the community composition of the nitrogen-fixing bacteria across four regions was evaluated through Canonical Correspondence Analysis (CCA) at the genus level, integrating soil physical and chemical factors with OTU data (Fig 4). The results revealed a significant correlation between TP content and the nitrogen-fixing bacterial community composition (*P* < 0.05). Meanwhile, in terms of projection of physical factors, the relative length associated with TP was the longest, followed by pH, whereas TN had the shortest. TP was determined to be the principal environmental factor shaping the community structure of the nitrogen-fixing bacteria, followed by pH and soil TN content. GJ4 was mainly projected onto the positive half-axis of TN, while MZ3 was projected onto the positive half-axis of TP. TN had a more significant impact on the community composition of the nitrogen-fixing bacteria in SRD. Based on a comprehensive analysis of the heatmap and CCA, it is concluded that the degree of rocky desertification, TN, TP, and pH are the primary factors influencing the community composition of the nitrogen-fixing bacteria in *V. villosa* root nodules within rocky desertification areas.

## Discussion

Rocky desertification, a significant ecological issue particularly in karst regions, refers to the process of land degradation characterized by the exposure of bedrock and the formation of rocky surfaces due to soil erosion and vegetation loss. This process is primarily driven by natural factors such as the region’s geological and climatic conditions, which include the presence of soluble rocks, high rainfall intensity, and a warm climate that accelerates rock dissolution. Additionally, human activities, including deforestation, overgrazing, and inappropriate agricultural practices, further exacerbate the problem. These combined factors result in sparse vegetation cover, widespread soil erosion, and severe ecological degradation. In such harsh environments, microbes play a crucial role. Nitrogen-fixing bacteria, which reside within plant tissues, show variations in species and functions across different plant species and environmental conditions. Nitrogen-fixing bacteria can enhance the growth of host plants [48], aiding them in tolerating stress conditions [49–51]. Nitrogen-fixing bacteria from arid regions can enhance the host plants’ resilience to salt and drought, as well as promote the growth of non-host crops under stress [52,53]. Therefore, studying the nitrogen-fixing bacteria within plants from rocky desertification areas is imperative.

In our investigation, Significant differences exist in soil physicochemical properties among different regions, and the communities within *V. villosa* nodules varied significantly across the four rocky desertification areas of southwest China. Moreover, the diversity of the nitrogen-fixing bacteria within these nodules was abundant. Compared to areas with MRD, the community composition and biodiversity in SRD were significantly higher. An increasing body of research highlights the vast diversity of microorganisms in arid and desert regions, capable of adapting to their surroundings and enduring harsh conditions [54]. The extent of rocky desertification influences the diversity of the nitrogen-fixing bacteria associated with plants. The composition of the nitrogen-fixing bacteria associated with *V. villosa* varies across different levels of rocky desertification.

The nitrogen-fixing bacteria in rocky desertification areas are crucial for helping plants adapt to harsh environmental conditions like drought, high temperatures, and infertile soils. Forming symbiotic relationships with plant roots, these bacteria improve plant absorption and utilization of water and nutrients, aiding in coping with adverse conditions. Additionally, these bacteria contribute to nitrogen cycling in the soil, thereby enhancing soil fertility. Moreover, they aid in the restoration of local vegetation and promote the overall health of the ecosystem. Research has shown that nitrogen-fixing bacteria can protect plants, such as eggplants, from the detrimental effects of drought stress [55]. Previous studies have shown that nitrogen-fixing bacteria can play a positive role in vegetation restoration and ecological improvement by enhancing soil fertility and promoting plant growth. Especially in karst rocky desertification areas, the presence of nitrogen-fixing bacteria may help improve the availability of soil nitrogen, thereby promoting the recovery of vegetation [56,57].Nitrogen-fixing bacteria have important potential application prospects in the restoration and management of rocky desertification ecosystems. Nitrogen-fixing bacteria play a significant role in improving soil fertility, restoring vegetation, and enhancing ecological stability. The application of nitrogen-fixing bacteria can serve as a sustainable land management strategy, reducing reliance on chemical fertilizers and lowering the risk of land degradation [58].

In this study, the community composition of the nitrogen-fixing bacteria in *V. villosa* nodules across four rocky desertification sites was significantly different. Within the same area, a negative correlation was observed between the diversity of the nitrogen-fixing bacteria and the degree of rocky desertification. Specifically, the lower the degree of rocky desertification was, the greater the diversity of the nitrogen-fixing bacteria was. All samples contained a significant amount of the major phylum Proteobacteria, and the distribution of this phylum varied significantly across the various regions. Proteobacteria, Acidobacteria, Bacteroidetes, and Actinobacteria were found to be the most abundant bacteria in the semi-arid Horqin Sandy terrain [59], similar to our study. The *nifH* gene was identified in previous studies examining the genetic diversity and abundance of culturable nitrogen-fixing bacteria in the maize phyllosphere of arid and semi-arid regions. Approximately 31.82% of the 242 isolated strains contained N-fixing genes and were classified under the phyla Firmicutes, Proteobacteria, Actinobacteria, and Bacteroidetes [60]. The composition of the nitrogen-fixing bacteria in *V. villosa* varied across different types of rocky desertification areas.

Furthermore, *Rhizobium*, with its nitrogen-fixing ability, can adapt to harsh rocky desertification areas. Studies have shown that *Pseudomonas* increases the content of terpenoids in *Cinnamomum camphora* [61]. *Rhizobium*, *Pseudomonas*, *Azospira*, and *Bacillus* can enhance above-ground and underground biomass, positively impacting crops [62]. Furthermore, in our study, the distribution of Enterobacteriaceae in SRD is high. Conversely, in MRD, the distribution of Enterobacteriaceae is very rare. This could be attributed to the lower stress resistance of the Enterobacteriaceae family, or it might be associated with bacterial symbiosis. Recent studies suggest that *ginger* rhizome development may be linked to the coexistence of Enterobacteriaceae and Pseudomonadaceae [63]. Enterobacteriaceae may also facilitate the decomposition of organic matter [64].

A multitude of environmental and host-related factors have the potential to exert an impact on the microbial community associated with plant hosts. These factors encompass geographical location, soil physicochemical properties, seasonality, plant phenotypes, and genotypes, as well as growth period [65]. This study also found that soil physicochemical properties have a significant impact on endogenous nitrogen-fixing bacteria. This investigation conducted in this study regarding the interactions between nitrogen-fixing bacteria and environmental factors has led to the following conclusions: The principal environmental factors that are likely to shape the community composition of nitrogen-fixing bacteria in *V. villosa*, are the degree of rocky desertification, TN, TP, and pH. Previous research has demonstrated that the introduction of exogenous *Sonneratia apetala* remarkably enhances the TN and alters the composition and structure of the nitrogen-fixing bacterial community [63]. A study focusing on the richness, grouping, and co-occurrence patterns of nitrogen-fixing bacterial communities in karst rocky desertification areas in southwest China discovered that soil pH, TN, and soil moisture can jointly influence bacterial community structure [66–68]. In our study, as the SWC in the rhizosphere of *V. villosa* increased, the degree of rocky desertification decreased, and concurrently, the richness and diversity of nitrogen-fixing bacteria in the plant's nodules increased. Consistent with other reports, our study revealed that different forms of Karst rocky desertification significantly affect the microbial community [69]. The influence of TN on microbial communities is more pronounced in the SRD area than in the MRD area. Finally, we detected a substantially positive correlation between the degree of rocky desertification and the dominant genus *Rhizobium*, which was inversely correlated with SWC in a significant manner. The degree of rocky desertification had the most significant effect on the community composition of nitrogen-fixing bacteria. Plant-associated nitrogen-fixing bacteria play a crucial role in improving soil quality, promoting vegetation restoration, and managing the ecological environment in rocky desertification areas. Nevertheless, the current application technologies for nitrogen-fixing bacteria remain immature. Therefore, further in-depth research is requisite to understand their mechanisms in soil improvement and vegetation restoration, to identify more effective application strategies. Additionally, other potential environmental variables, such as soil organic matter content, interactions among soil microorganisms, and changes in precipitation, may also significantly influence nitrogen-fixing bacterial communities [70]. These factors could play important roles in shaping bacterial community structure, highlighting their significance in future research. Although our research results are statistically significant at the current sample size, in order to further verify these findings, future studies need to expand the sample size. This will help improve the robustness and generalizability of the research results. Future research could further explore the long-term effects of different environmental variables on the community structure of nitrogen-fixing bacteria and validate our findings across a broader geographical range. Additionally, employing more advanced molecular techniques might provide deeper insights into how nitrogen-fixing bacteria respond to environmental changes at the molecular level [71]. Furthermore, land resources in rocky desertification areas are severely degraded, and the application of nitrogen-fixing bacteria should be integrated with other land management practices to attain more favorable outcomes.

## Conclusions

In the present study, a comprehensive analysis was carried out to investigate the diversity and community structure of nitrogen-fixing bacteria in the root nodules of *V. villosa* sourced from four rocky desertification areas. Additionally, the physicochemical properties of rhizosphere soil were also examined. The results of our research work indicated that there were significant differences in the bacterial community composition among the *V. villosa* samples obtained from Jianshui, Gejiu, and Mengzi in Southwest China. Notably, Rhizobium emerged as the dominant nitrogen-fixing bacteria across all four rocky desertification areas under consideration. Probably, the degree of rocky desertification, along with the levels of TN, TP, and pH constitute the primary environmental factors that have a significant influence on the composition of the nitrogen-fixing bacterial community in *V. villosa*. The findings of this study offer valuable and profound insights into the intricate relationships between nitrogen-fixing bacteria and environmental factors. Looking ahead, it is anticipated that by augmenting the levels of soil TN, TP, and pH, the diversity of the nitrogen-fixing bacterial community could be enhanced. This, in turn, might potentially lead to an increase in the diversity of other species within rocky desertification areas and thereby contribute to the effective control and mitigation of rocky desertification.

## Supporting information

S1 Table Basic information of four rocky desertification plot samples in the study: Gejiu1 (GJ1), Gejiu4 (GJ4), Mengzi3 (MZ3), Kaiyuan2 (KY2), slight rocky desertification (SRD), and moderate rocky desertification (MRD). (DOCX)

S2 Table Statistical analysis results of soil physical and chemical properties under different degrees of rocky desertification. (DOCX)

S3 Table Statistical Results of α diversity analysis of nitrogen-fixing bacteria in *V. villosa.* (DOCX)

## References

[1] Wang S, Li Y, Li R. Karst rocky desertification: formation background, evolution and comprehensive taming. Quaternary Sciences. 2003;(06):657–66.

[2] Liu F, Wang S, Liu Y, He T, Luo H, Long J. Changes of soil quality in the process of karst rocky desertification and evaluation of impact on ecological environment. Acta Ecologica Sinica. 2005;(03):639–44.

[3] Qin X, Zhu M, Jiang Z. A review on recent advances in rocky desertification in southwest China karst region. Carsologica Sinica. 2006;(03):234–8.

[4] Gu Y, Chen F, Li K, Wu H. Ecological control of land rocky desertification and vegetation recovery in karst region of Yunnan Province. Sci Technol Review. 2009;27(05):75–80.

[5] Liu L, He X, Du H, Wang K. The relationships among nitrogen-fixing microbial communities, plant communities, and soil properties in karst regions. Acta Ecologica Sinica. 2017;37(12):4037–44.

[6] Zhang Y, Zhou Y, Chang E. The problem of desertification in Yunnan Province. Forestry economics. 2010;(05): 72–4.

[7] Lu Z-X, Wang P, Ou H-B, Wei S-X, Wu L-C, Jiang Y, et al. Effects of different vegetation restoration on soil nutrients, enzyme activities, and microbial communities in degraded karst landscapes in southwest China. Forest Ecol Management. 2022;508:120002. doi: 10.1016/j.foreco.2021.120002

[8] Guan H, Fan J. Effects of vegetation restoration on soil quality in fragile karst ecosystems of southwest China. PeerJ. 2020;8:e9456. doi: 10.7717/peerj.9456 32676227 PMC7335502

[9] Lu X, Yang W, Xi Y, Ding F. Effects of different vegetation recovery types on soil chemical and microbial biomass properties in Maolan Karst region. J Nanjing Forestry University (Natural Sciences Edition). 2015;39(05):73–80.

[10] Pingjiu Z, Lianqing L, Genxing P, Jingchen R. Soil quality changes in land degradation as indicated by soil chemical, biochemical and microbiological properties in a karst area of southwest Guizhou, China. Environ Geol. 2006;51(4):609–19.

[11] Tang Q, Li Q, Tong L, Wu R, Xu J. Rhizospheric soil organic carbon accumulated but its molecular groups redistributed via rhizospheric soil microorganisms along multi-root Cerasus humilis plantation chronosequence at the karst rocky desertification control area. Environ Sci Pollut Res Int. 2023;30(28):72993–3007. doi: 10.1007/s11356-023-27588-9 37184792

[12] Zheng W, Wu Q, Rao C, Chen X, Wang E, Liang X, et al. Characteristics and interactions of soil bacteria, phytocommunity and soil properties in rocky desertification ecosystems of Southwest China. CATENA. 2023;220:106731. doi: 10.1016/j.catena.2022.106731

[13] Dai Y, Zang L, Zhang G, Liu Q, Sui M, He Y, et al. The composition and functional roles of soil autotrophic microorganisms in vegetation restoration of degraded karst forest. European J Forest Res. 2024;143(6):1701–15.

[14] Guillermo BC, José A, Daniel G, L AC, David B. Occurrence and intensity of wild boar disturbances, effects on the physical and chemical soil properties of alpine grasslands. Plant and Soil. 2013;373(1–2):243–56.

[15] Wu H, Liu Y, Zhang T, Xu M, Rao B. Impacts of Soil Properties on Species Diversity and Structure in Alternanthera philoxeroides-Invaded and Native Plant Communities. Plants (Basel). 2024;13(9):1196. doi: 10.3390/plants13091196 38732411 PMC11085794

[16] Wen D, Huang Y, Huang Y, Ding N, Ni K, Wang H, et al. Karst rocky desertification restoration increases soil inorganic N supply to reduce plant N limitation. CATENA. 2024;241:108012. doi: 10.1016/j.catena.2024.108012

[17] Liang Y-M, Su Y-R, He X-Y, Chen X-B. Effects of Lithology on the Abundance and Composition of Soil Nitrogen-fixing Bacteria and Arbuscular Mycorrhizal Fungal Communities in Karst Shrub Ecosystem. Huan Jing Ke Xue. 2017;38(3):1253–61. doi: 10.13227/j.hjkx.201606215 29965601

[18] Tang C, Gao R, Tang X, Zhang Y, Feng W, Feng B, et al. Metabolites isolated from Penicillium HDS-Z-1E, an endophytic fungal strain isolated from Taxus cuspidata and their activation effect of catalase. Chin Herb Med. 2023;16(2):227–30. doi: 10.1016/j.chmed.2022.12.009 38706817 PMC11064617

[19] Feng Y, Shen D, Song W. Rice endophyte Pantoea agglomerans YS19 promotes host plant growth and affects allocations of host photosynthates. J Appl Microbiol. 2006;100(5):938–45. doi: 10.1111/j.1365-2672.2006.02843.x 16629994

[20] Liu T, Zhai C, Zhang J, Coulter JA. Genetic diversity and promotion plant growth of culturable endophytic diazotrophs associated with seashore paspalum cultivars. New Zealand J Crop and Horticultural Science. 2021;49(2–3):243–57. doi: 10.1080/01140671.2021.1893193

[21] Kurrey DK, Sharma R, Lahre MK, Kurrey RL. Effect of Azotobacter on physio-chemical characteristics of soil in onion field. AkiNik Publications. 2018;7(2):108–13.

[22] Sumbul A, Ansari RA, Rizvi R, Mahmood I. Azotobacter: A potential bio-fertilizer for soil and plant health management. Saudi J Biol Sci. 2020;27(12):3634–40. doi: 10.1016/j.sjbs.2020.08.004 33304174 PMC7714982

[23] Mendoza-Suárez MA, Geddes BA, Sánchez-Cañizares C, Ramírez-González RH, Kirchhelle C, Jorrin B, et al. Optimizing Rhizobium-legume symbioses by simultaneous measurement of rhizobial competitiveness and N2 fixation in nodules. Proc Natl Acad Sci U S A. 2020;117(18):9822–31. doi: 10.1073/pnas.1921225117 32317381 PMC7211974

[24] Lopes ÁL de O, Setubal IS, Costa Neto VP da, Zilli JE, Rodrigues AC, Bonifacio A. Synergism of Bradyrhizobium and Azospirillum baldaniorum improves growth and symbiotic performance in lima bean under salinity by positive modulations in leaf nitrogen compounds. Appl Soil Ecol. 2022;180:104603. doi: 10.1016/j.apsoil.2022.104603

[25] Pan L, Liang C, Liu Y. Study on the ecological and efficient planting mode of Rubus chingii-Viciavillosa rothvar-Apis cerana in Jingning Mountain Area. Yunnan Agricultural Sci Technology. 2022;(03):25–7.

[26] Deguchi S, Uchino H, Uozumi S, Touno E, Morita S. The inorganic nitrogen fertilizer equivalency of hairy vetch ( *Vicia villosa* Roth) as a winter green manure in Kanto and Tohoku district, Japan. Soil Science and Plant Nutrition. 2022;68(2):268–74. doi: 10.1080/00380768.2022.2037392

[27] Zhao W, Xue K, Yang J, Hu B, Fu L, Yin M. Effects of various amounts of smooth vetch on yield and quality of tobacco under reduction of nitrogen fertilizer. Jiangsu Agricultural Sciences. 2022;50(16):73–8.

[28] Nong Q, Lin L, Xie J, Mo Z, Malviya MK, Solanki MK, et al. Regulation of an endophytic nitrogen-fixing bacteria GXS16 promoting drought tolerance in sugarcane. BMC Plant Biol. 2023;23(1):573. doi: 10.1186/s12870-023-04600-5 37978424 PMC10655487

[29] Ji H, Pan C, Zhou J, Luo M, Wang W, Luo D. Adaptability of endophytic diazotrophic bacteria strains from desert shrubs to diverse environment factors. J Desert Res. 2011;31(04):942–7.

[30] Zhu MZ. Resource of symbiotic nitrogen-fixation of legume and diversity of Rhizobia in karst shrub communities. Central South University of Forestry and Technology. 2012.

[31] Kong D, Qu L, Ren X, Gui W. Status and ecological restoration strategy of rocky desertification land in Yunnan Province based on ecological space protection. Forest Inventory and Planning. 2022;47(04):160–5.

[32] Zhao L, Lei Y, Chen J, Zhu S, Zhou J, Tang F. Evolution process and comprehensive control of rocky desertification in Honghe state, Yunnan Province. Carsologica sinica. 2019;38(05):704–12.

[33] Gu L, Duan T. Current status of research on *Vicia villosa* var. glabresens as indicated by a bibliometric analysis using the China National Knowledge Infrastructure (CNKI) database. Acta Prataculturae Sinica. 2021;30(11):221–8.

[34] Puri A, Padda KP, Chanway CP. Can naturally-occurring endophytic nitrogen-fixing bacteria of hybrid white spruce sustain boreal forest tree growth on extremely nutrient-poor soils?. Soil Biology and Biochemistry. 2020;140:107642. doi: 10.1016/j.soilbio.2019.107642

[35] Wang C, Lei J, Jin X, Cai S, Zou Y, Sun X. Isolation, identification and phylogenetic analysis of endophytic nitrogen-fixing bacteria from sweet potato tuberous root. J Southern Agriculture. 2023;54(05):1397–404.

[36] Feng Q, Gao J, Yan S, Wang J, Liang C, Qin H. Characteristics and diversity of endophytic diazotrophs in three bamboo species. J Zhejiang A&F University. 2021;38(06):1203–12.

[37] Ferrando L, Rariz G, Martínez-Pereyra A, Fernández-Scavino A. Endophytic diazotrophic communities from rice roots are diverse and weakly associated with soil diazotrophic community composition and soil properties. J Appl Microbiol. 2024;135(7):lxae157. doi: 10.1093/jambio/lxae157 38925647

[38] Bai L, Yang Y, Shi Z, Zou Y, Zhou H, Jia J. Improvement of Low-Fertility Soils from a Coal Mining Subsidence Area by Immobilized Nitrogen-Fixing Bacteria. Processes. 2022;10(6):1185. doi: 10.3390/pr10061185

[39] Dai QH, Yan YJ. Research Progress of Karst Rocky Desertification and Soil Erosion in Southwest China. Bulletin of Soil and Water Conservation. 2018;32(02):1–10.

[40] Li RL, Wang SJ, Xiong KN, Zhou DQ. Correlation Between Rocky Desertification and Slope Degree in Karst Area of Guizhou. Bulletin of Soil and Water Conservation. 2006;(04):82–6.

[41] Cao H, Xu L, Song J, Xun M, Zhang W, Yang H. Bacterial community structure and co-occurrence networks in the rhizosphere and root endosphere of the grafted apple. BMC Microbiol. 2024;24(1). doi: 10.1186/s12866-024-03210-x PMC10858598 38341527

[42] Ding Y, Xiong Z, Wang M, Liu S, Xie E, Wang J. Diversity analysis of endophytic bacterial separated from the leguminous plants nodules of Wenshan rocky desertification areas. J Southern Agriculture. 2015;46(04):602–8.

[43] Yu Y, Chen Z, Xie H, Feng X, Wang Y, Xu P. Overhauling the Effect of Surface Sterilization on Analysis of Endophytes in Tea Plants. Front Plant Sci. 2022;13:849658. doi: 10.3389/fpls.2022.849658 35592578 PMC9111953

[44] Su B, Zhang Y, Dao R. Study on the distribution characteristics of bacterial communities in the rhizosphere soil of four leguminous cultivated forages. Acta Prataculturae Sinica. 2021;29(02):250–8.

[45] Pedrolo AM, Matteoli FP, Soares CRFS, Arisi ACM. Comparative Genomics Reveal the High Conservation and Scarce Distribution of Nitrogen Fixation nif Genes in the Plant-Associated Genus Herbaspirillum. Microb Ecol. 2023;86(1):563–74. doi: 10.1007/s00248-022-02084-8 35932316

[46] Yang Y, Li Y, Hao K, Zhao Y, Li M, Fan Y. Microbial community composition and co-occurrence network analysis of the rhizosphere soil of the main constructive tree species in Helan Mountain of Northwest China. Sci Rep. 2024;14(1):24557. doi: 10.1038/s41598-024-76195-2 39427091 PMC11490567

[47] Schloss PD, Westcott SL, Ryabin T, Hall JR, Hartmann M, Hollister EB, et al. Introducing mothur: open-source, platform-independent, community-supported software for describing and comparing microbial communities. Appl Environ Microbiol. 2009;75(23):7537–41. doi: 10.1128/AEM.01541-09 19801464 PMC2786419

[48] Rosenblueth M, Martínez-Romero E. Bacterial endophytes and their interactions with hosts. Mol Plant Microbe Interact. 2006;19(8):827–37. doi: 10.1094/MPMI-19-0827 16903349

[49] Peng H, de-Bashan LE, Higgins BT. Comparison of algae growth and symbiotic mechanisms in the presence of plant growth promoting bacteria and non-plant growth promoting bacteria. Algal Res. 2021;53:102156. doi: 10.1016/j.algal.2020.102156

[50] Din I, Khan H, Ahmad Khan N, Khil A. Inoculation of nitrogen fixing bacteria in conjugation with integrated nitrogen sources induced changes in phenology, growth, nitrogen assimilation and productivity of wheat crop. J Saudi Soc Agricultural Sci. 2021;20(7):459–66. doi: 10.1016/j.jssas.2021.05.008

[51] Sharma C, Sharma P, Kumar A, Walia Y, Kumar R, Umar A. A review on ecology implications and pesticide degradation using nitrogen fixing bacteria under biotic and abiotic stress conditions. Chemistry Ecol. 2023;39(7):753–74.

[52] Wanore DS, Bachore MA, Eromo EK, Nure RK, Bidiko GB, Abdo SS. Isolation and Characterization of Nitrogen Fixing Bacteria from Rhizospher of Chickpea and Peanodules, Hossana, Ethiopia. UPJOZ. 2023;44(15):97–107. doi: 10.56557/upjoz/2023/v44i153574

[53] Salmasi SZ, Nasiri H, Heshmati R, Sarikhani MR, Raei Y. The impact of nitrogen-fixing bacteria, iron, and zinc foliar application on dry land yellow mustard (Brassica juncea) grain and oil production. Agricultural Sciences. 2024;15(07):719–28.

[54] Mapelli F, Marasco R, Balloi A, Rolli E, Cappitelli F, Daffonchio D, et al. Mineral-microbe interactions: biotechnological potential of bioweathering. J Biotechnol. 2012;157(4):473–81. doi: 10.1016/j.jbiotec.2011.11.013 22138043

[55] Kiran S, Furtana GB, Ellialtioglu ŞŞ. Physiological and biochemical assay of drought stress responses in eggplant (Solanum melongena L.) inoculated with commercial inoculant of Azotobacter chroococum and Azotobacter vinelandii. Scientia Horticulturae. 2022;305:111394. doi: 10.1016/j.scienta.2022.111394

[56] Rongxiao C, Yongcui D, Yibo W, Jing Z, Fang W, Li T. Relationships between biological nitrogen fixation and available nitrogen at scales from molecular to community level. Chinese J Ecol. 2017;36(01):224–32. doi: 10.13292/j.1000-4890.201701.009

[57] Li J. Changes of microbiome closely associated with plants after topsoil transfer. Yunnan University. 2022.

[58] Suleiman MK, Quoreshi AM, Bhat NR, Manuvel AJ, Sivadasan MT. Divulging diazotrophic bacterial community structure in Kuwait desert ecosystems and their N2-fixation potential. PLoS One. 2019;14(12):e0220679. doi: 10.1371/journal.pone.0220679 31877136 PMC6932743

[59] Zhang Y, Cao C, Peng M, Xu X, Zhang P, Yu Q, et al. Diversity of nitrogen-fixing, ammonia-oxidizing, and denitrifying bacteria in biological soil crusts of a revegetation area in Horqin Sandy Land, Northeast China. Ecological Engineering. 2014;71:71–9. doi: 10.1016/j.ecoleng.2014.07.032

[60] Abadi VAJM, Sepehri M, Rahmani HA, Dolatabad HK, Shamshiripour M, Khatabi B. Diversity and abundance of culturable nitrogen-fixing bacteria in the phyllosphere of maize. J Appl Microbiol. 2021;131(2):898–912. doi: 10.1111/jam.14975 33331107

[61] Zhang G-F, Huang Q-L, Bi X-Q, Liu Y-L, Yuan Z-S. Analysis of endophytic bacterial community diversity and metabolic correlation in Cinnamomum camphora. Arch Microbiol. 2020;202(1):181–9. doi: 10.1007/s00203-019-01733-w 31562551

[62] Igiehon NO, Babalola OO. Rhizosphere Microbiome Modulators: Contributions of Nitrogen Fixing Bacteria towards Sustainable Agriculture. Int J Environ Res Public Health. 2018;15(4):574. doi: 10.3390/ijerph15040574 29570619 PMC5923616

[63] Huang K, Sun X, Zou Y, Li H, Xu P, Zhang W, et al. Comparison of the endophytic bacterial microbiota of asymptomatic and symptomatic ginger rhizomes during the activation of adventitious bud development. Plant disease. 2022;106(9). doi: 10.1094/pdis-09-21-2069-re 35286131

[64] Fu Y, Ding C, Fan J, Li Y, Yao L, Yang M, et al. Effects of three regeneration methods on the growth and bacterial community diversity of Populus × euramericana. PLoS One. 2022;17(8):e0273306. doi: 10.1371/journal.pone.0273306 36018851 PMC9416986

[65] Hamonts K, Trivedi P, Garg A, Janitz C, Grinyer J, Holford P, et al. Field study reveals core plant microbiota and relative importance of their drivers. Environ Microbiol. 2018;20(1):124–40. doi: 10.1111/1462-2920.14031 29266641

[66] Yang L. Effects of vegetation recovery on diazotrophs community and nitrogenase activity in karst areas of Guizhou Province. Shanxi Normal University. 2020.

[67] Xiao D, Hong T, Chen M, He X, Wang K. Assessing the Effect of Slope Position on the Community Assemblage of Soil Diazotrophs and Root Arbuscular Mycorrhizal Fungi. J Fungi (Basel). 2023;9(4):394. doi: 10.3390/jof9040394 37108849 PMC10145487

[68] Liang Y, He X, Chen X, Su Y, Pan F, Hu L. Low Frequency of Plants Associated with Symbiotic Nitrogen-Fixers Exhibits High Frequency of Free-Living Nitrogen Fixing Bacteria: A Study in Karst Shrub Ecosystems of Southwest China. Forests. 2022;13(2):163. doi: 10.3390/f13020163

[69] Cao W, Xiong Y, Zhao D, Tan H, Qu J. Bryophytes and the symbiotic microorganisms, the pioneers of vegetation restoration in karst rocky desertification areas in southwestern China. Appl Microbiol Biotechnol. 2020;104(2):873–91. doi: 10.1007/s00253-019-10235-0 31822979 PMC6943408

[70] Sharma S, Singh DK. Temporal Variations in Diazotrophic Communities and nifH Transcripts Level Across the Agricultural and Fallow Land at Jaipur, Rajasthan, India. Indian J Microbiol. 2017;57(1):92–9. doi: 10.1007/s12088-016-0634-0 28148984 PMC5243255

[71] Soares FS, Rangel de Souza ALS, de Souza SA, de Souza Vespoli L, Pinto VB, Matiello L, et al. Fine-Tuning of Arabidopsis thaliana Response to Endophytic Colonization by Gluconacetobacter diazotrophicus PAL5 Revealed by Transcriptomic Analysis. Plants (Basel). 2024;13(13):1719. doi: 10.3390/plants13131719 38999559 PMC11244368

