# Supplementary material for: Relationships between nitrogen-fixing bacteria community structure in Vicia villosa nodules, soil properties and rocky desertification degree in karst area southwest China
Source: PLoS One. 2025 Aug 1;20(8):e0329408. doi: 10.1371/journal.pone.0329408 (PMC12316310; doi:10.1371/journal.pone.0329408)
Supplement: S1 Table — (DOCX) [file pone.0329408.s001.docx]

**Table S1.** Basic information of four rocky desertification plot samples in the study: Gejiu1 (GJ1), Gejiu4 (GJ4), Mengzi3 (MZ3), Kaiyuan2 (KY2), slight rocky desertification (SRD), and moderate rocky desertification (MRD)

| Plot areas | Plot locations | Disturbance conditions | Rocky desertification degree  (RD) | Longitude and latitude | Altitude (m) |
| --- | --- | --- | --- | --- | --- |
| GJ1 | Yangjiatian Village, Xicheng Town, Gejiu City, Yunnan Province | Deserted field without disturbance | MRD | N 23°21′11″  E 103°9′1″ | 1797.77 |
| GJ4 | Wugu Shao Village, Xicheng Town, Gejiu City, Yunnan Province | Deserted field without disturbance | SRD | N 23°19′28″  E 103°9′58″ | 1896.14 |
| MZ3 | Zhuangzhai Village, Zhicun Town, Mengzi City, Yunnan Province | Deserted field without disturbance | MRD | N 23°21′21″  E 103°31′13″ | 1624.87 |
| KY2 | Zhumashao Village, Dazhuang Town, Kaiyuan City, Yunnan Province | Deserted field without disturbance | MRD | N 23°37′20″  E 103°18′29″ | 1310.42 |

Note: RD (Rocky Desertification Degree): A measure of the extent of rocky desertification, with MRD indicating Moderate Rocky Desertification and SRD indicating Slight Rocky Desertification.
