# Supplementary material for: Relationships between nitrogen-fixing bacteria community structure in Vicia villosa nodules, soil properties and rocky desertification degree in karst area southwest China
Source: PLoS One. 2025 Aug 1;20(8):e0329408. doi: 10.1371/journal.pone.0329408 (PMC12316310; doi:10.1371/journal.pone.0329408)
Supplement: S2 Table — (DOCX) [file pone.0329408.s002.docx]

**Table S2.** Statistical analysis results of soil physical and chemical properties under different degrees of rocky desertification

| Environmental factor | F values | The degreens of freedom | The precise p values |
| --- | --- | --- | --- |
| pH | 2.146 | 3 | 0.173 |
| TN | 20.805 | 3 | 0.000 |
| TP | 16.174 | 3 | 0.001 |
| AN | 30.610 | 3 | 0.000 |
| AP | 169.815 | 3 | 0.000 |
| SWC | 5.968 | 3 | 0.019 |
| AK | 51.533 | 3 | 0.000 |
| TK | 5.889 | 3 | 0.020 |

Legend description: This table presents the statistical analysis results of soil physical and chemical properties under different degrees of rocky desertification. The F-value, degrees of freedom and exact P-value were calculated through Analysis of Variance (ANOVA) to evaluate the influence of different degrees of rocky desertification on the physical and chemical properties of the soil.
