# Supplementary material for: Relationships between nitrogen-fixing bacteria community structure in Vicia villosa nodules, soil properties and rocky desertification degree in karst area southwest China
Source: PLoS One. 2025 Aug 1;20(8):e0329408. doi: 10.1371/journal.pone.0329408 (PMC12316310; doi:10.1371/journal.pone.0329408)
Supplement: S3 Table — (DOCX) [file pone.0329408.s003.docx]

**Table S3.** Statistical Results of α diversity analysis of nitrogen-fixing bacteria in *V. villosa.*

| diversity index | F values | The degreens of freedom | The precise p values |
| --- | --- | --- | --- |
| sobs | 2.803 | 3 | 0.108 |
| shannon | 2.394 | 3 | 0.144 |
| simpson | 3.374 | 3 | 0.075 |
| ace | 1.302 | 3 | 0.339 |
| chao | 3.142 | 3 | 0.087 |
| coverage | 0.401 | 3 | 0. 756 |

Legend description: This table presents the statistical analysis results of the α-diversity index of nitrogen-fixing bacteria in *V. villosa.* The F-value, degrees of freedom, and exact p-value were calculated through Analysis of Variance (ANOVA) to evaluate the impact of varying degrees of stone desertification on the α-diversity of nitrogen-fixing bacteria in *V. villosa.*
